# Embedding a membrane protein into an enveloped artificial viral replica†

**DOI:** 10.1039/d1cb00166c

**Published:** 2021-12-21

**Authors:** Hiroto Furukawa, Hiroshi Inaba, Yoshihiro Sasaki, Kazunari Akiyoshi, Kazunori Matsuura

**Affiliations:** Department of Chemistry and Biotechnology, Graduate School of Engineering, Tottori University Koyama-Minami 4-101 Tottori 680-8552 Japan ma2ra-k@tottori-u.ac.jp; Centre for Research on Green Sustainable Chemistry, Tottori University Koyama-Minami 4-101 Tottori 680-8552 Japan; Department of Polymer Chemistry, Graduate School of Engineering, Kyoto University Katsura Nishikyo-ku Kyoto 615-8510 Japan

## Abstract

Natural enveloped viruses, in which nucleocapsids are covered with lipid bilayers, contain membrane proteins on the outer surface that are involved in diverse functions, such as adhesion and infection of host cells. Previously, we constructed an enveloped artificial viral capsid through the complexation of cationic lipid bilayers onto an anionic artificial viral capsid self-assembled from β-annulus peptides. Here we demonstrate the embedding of the membrane protein Connexin-43 (Cx43), on the enveloped artificial viral capsid using a cell-free expression system. The expression of Cx43 in the presence of the enveloped artificial viral capsid was confirmed by western blot analysis. The embedding of Cx43 on the envelope was evaluated by detection *via* the anti-Cx43 antibody, using fluorescence correlation spectroscopy (FCS) and transmission electron microscopy (TEM). Interestingly, many spherical structures connected to each other were observed in TEM images of the Cx43-embedded enveloped viral replica. In addition, it was shown that fluorescent dyes could be selectively transported from Cx43-embedded enveloped viral replicas into Cx43-expressing HepG2 cells. This study provides a proof of concept for the creation of multimolecular crowding complexes, that is, an enveloped artificial viral replica embedded with membrane proteins.

## Abstract 2

We demonstrate the embedding membrane protein, Cx43, on the enveloped artificial viral capsid using a cell-free expression system. The embedding of Cx43 on the envelope was evaluated by detection with anti-Cx43 antibody using FCS and TEM.

## Graphical abstract

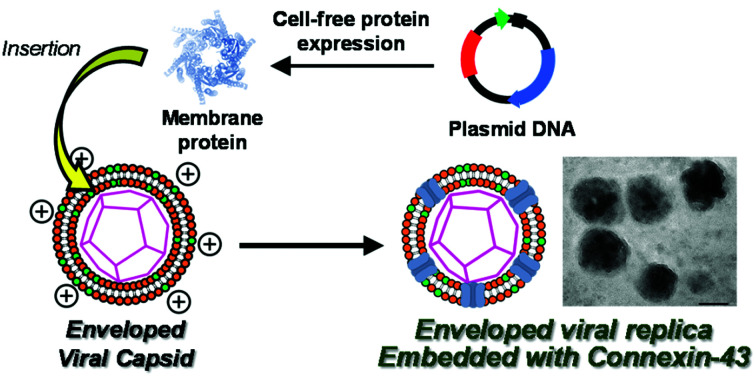

## Introduction

Enveloped viruses such as the influenza virus, human immunodeficiency virus, and coronavirus are nano-sized multimolecular crowding complexes consisting of nucleocapsids covered by a lipid bilayer.^1–3^ Membrane proteins embedded in the outer surface of enveloped viruses are involved in diverse functions, including adhesion and infection of host cells. For example, spike proteins embedded in the envelope of the coronavirus play an important role during host cell infection.^4,5^ The influenza virus has two different membrane proteins, haemagglutinin and neuraminidase, on its envelope, which are involved in infection and budding, respectively.^6,7^

Over the past two decades, natural self-assembling protein nanocapsules, such as viral capsids,^8–16^ lumazine synthase,^17^ ferritin,^18^ carboxysome,^19^ clathrin,^20^ encapsulin,^21,22^ and their variants, have emerged as attractive organic materials of discrete size, unique morphology, and constant assembly number. Protein nanocapsules have been exploited as nanocarriers for drug delivery systems (DDSs), nanotemplates, and nanoreactors. Recently, artificial protein nanocapsules mimicking natural viral capsids have been actively developed by self-assembly of rationally designed proteins.^23–28^ For example, Baker *et al.* designed *de novo* artificial protein subunits with various symmetries and succeeded in constructing protein nanocapsules that are stable against denaturation reagents and heat.^27,28^ The *de novo*-designed protein nanocapsules were utilised as a scaffold for vaccine candidates. The display of the receptor binding domain of the spike protein from SARS-CoV-2 and haemagglutinin from the influenza virus on the surface of artificial protein nanocapsules activated antibody production by the immune system, and induced a strong neutralising antibody response.^29–32^ However, direct expression of membrane protein motifs on protein scaffolds may significantly affect the structure and properties of the nanocapsules and membrane proteins. If nano-architecture similar to natural enveloped viruses embedded with various functional membrane proteins can be readily constructed, it may be possible to overcome the previous challenges. To date, there have been no examples of construction of artificial enveloped viral capsids embedded with membrane proteins, which remains a challenging task.

On the other hand, the progressive development of nano-architectures, self-assembled from rationally designed peptides, have enabled the construction of viral capsid-like nanocapsules consisting of peptides.^33–40^ We previously found that a 24-residue β-annulus peptide (INHVGGTGGAIMAPVAVTRQLVGS), which is involved in the formation of the dodecahedral inner skeleton of the tomato bushy stunt virus, self-assembles spontaneously into a hollow artificial viral capsid with a size range of 30–50 nm in water.^36,37,41^ The artificial viral capsid can encapsulate various guest molecules inside and can be modified with functional molecules on the external surface.^36,41–45^ We recently succeeded in constructing an enveloped artificial viral capsid by complexing an anionic artificial viral capsid self-assembled from the β-annulus-EE peptide (INHVGGTGGAIMAPVAVTRQLVGSEE) with 1,2-dioleoyl-3-trimethylammonium-propane (DOTAP)/1,2-dioleoyl-*sn-glycero*-3-phosphocholine (DOPC) mixed lipids through electrostatic interaction.^46^ This enveloped virus-mimicking complex has a relatively uniform particle size and forms a stable structure, as the critical aggregation concentration is 100 times lower than that of the capsid alone, suggesting that it is a candidate for DDS carriers and artificial models of enveloped viruses.

Membrane protein-embedded liposomes (proteoliposomes) can be constructed by expressing membrane proteins using a cell-free protein expression system (PURE system) and simultaneously embedding them into liposomes.^47–51^ The PURE system synthesises the target proteins by mixing purified initiation factors, elongation factors, ribosome regeneration factors, necessary enzyme proteins, amino acids, NTPs, and tRNAs involved in the translation reaction in *E. coli*. The advantages of this method are that its composition can be freely modified and there are almost no proteins irrelevant to protein synthesis because it does not contain cell extracts, but only purified factors. Akiyoshi *et al.* demonstrated that Connexin-43 (Cx43), a membrane protein involved in the transport of substances between cells, can be embedded in liposomes using the PURE system.^50^ The fluorescent dye, calcein, encapsulated in the Cx43-embedded proteoliposomes may be transported into the cells through the gap junctions between Cx43 molecules.^50^

The question of whether functional membrane proteins can be embedded on the enveloped artificial capsid using the PURE system is unexplored. In this study, we constructed an enveloped artificial viral capsid embedded with the functional membrane protein, Cx43, using the PURE system (referred to as an enveloped artificial viral replica). The function of the embedded Cx43 on the enveloped capsid was evaluated by western blot analysis, fluorescence correlation spectroscopy (FCS), and transmission electron microscopy (TEM). In addition, the transport of fluorescent small dyes from the Cx43-embedded enveloped viral replica into Cx43-expressing HepG2 cells was evaluated by confocal laser scanning microscopy (CLSM). The results provide a new proof of concept for creating multimolecular crowding complexes, which are enveloped artificial viral replica embedded with membrane proteins.

## Results and discussion

### Expression of Cx43 in the presence of an enveloped artificial viral capsid

**Fig. 1:**
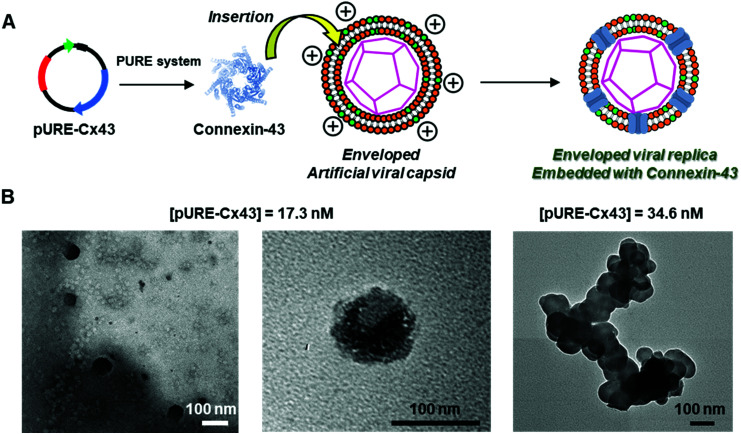
(A) Schematic illustration and (B) TEM images of enveloped viral capsid embedded with Cx43 ([β-annulus-EE] = 16 μM, [DOTAP] = 48 μM, [DOPC] = 480 μM). Cx43 was expressed from 17.3 to 34.6 nM pURE-Cx43 using the PURE system. The samples were stained with EM stainer.

To construct the enveloped artificial viral capsid containing Cx43, we employed a 26-residue β-annulus-EE peptide (INHVGGTGGAIMAPVAVTRQLVGSEE, *m*/*z* = 2563 [M]^+^, Fig. S1, ESI†) as the scaffold, which possesses two anionic Glu residues in the C-terminal region directed towards the outer surface of the capsid.^46^ The artificial viral capsid possessing an anionic surface was complexed with a mixture of a cationic lipid, DOTAP, and a zwitterionic lipid, DOPC, using a hydration method to construct an enveloped artificial viral capsid with a size of 84 ± 23 nm.^46^ Then, cell-free protein expression of the plasmid encoding Cx43 (pURE-Cx43) was conducted using the PURE system in the presence of the enveloped artificial viral capsid (Fig. 1A). The TEM image of the post-expression solution ([pURE-Cx43] = 17.3 nM), showed spherical structures of 50–100 nm (Fig. 1B), and the size distribution obtained from TEM images and dynamic light scattering (DLS) were 103 ± 46 nm and 58 ± 11 nm, respectively (Fig. S2 and S3A, ESI†). The difference in size distribution obtained from TEM and DLS might be caused by the difference in the observation conditions, that is, the particle size observed by TEM in the dry condition is likely to be large. On the other hand, at higher plasmid concentrations (34.6, 69.1 nM), aggregates of spherical assemblies of approximately 100 nm were primarily observed (Fig. 1B and Fig. S4, ESI†). The size distribution of the Cx43-expressed solution ([pURE-Cx43] = 34.6 nM) obtained from DLS was 26 ± 4 nm and 571 ± 122 nm (Fig. S3B, ESI†). The small particle sizes might be derived from the PURE system reagents, and the large ones from the aggregates of the spherical assemblies. Cx43 is a four-fold transmembrane protein that is involved in the transport of substances between cells. It forms a hexamer (connexon) on the cell membrane, which in turn forms a gap junction with the connexon of other cells.^52,53^ The aggregation reaction may be caused by the accumulation of increased amounts of Cx43 on the envelope and by the formation of excessive gap junction structures among the spherical structures.

**Fig. 2:**
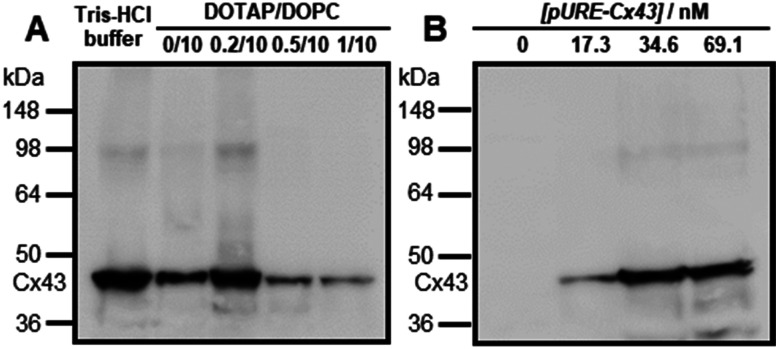
(A) Western blot analysis of Cx43 expressed from 17.3 nM pURE-Cx43 in the presence of enveloped viral capsid ([β-annulus-EE] = 16 μM, [DOPC] = 480 μM (DOTAP/DOPC = 0/10); [β-annulus-EE] = 16 μM, [DOTAP] = 9.6 μM, [DOPC] = 480 μM (DOTAP/DOPC = 0.2/10); [β-annulus-EE] = 16 μM, [DOTAP] = 24 μM, [DOPC] = 480 μM (DOTAP/DOPC = 0.5/10); [β-annulus-EE] = 16 μM, [DOTAP] = 48 μM, [DOPC] = 480 μM (DOTAP/DOPC = 1/10)) in 10 mM Tris–HCl buffer (pH 7.0). (B) Western blot analysis of the concentration dependence of pURE-Cx43 (0, 17.3, 34.6, 69.1 nM) in the presence of enveloped viral capsid ([β-annulus-EE] = 16 μM, [DOTAP] = 48 μM, [DOPC] = 480 μM (DOTAP/DOPC = 1/10)) in 10 mM Tris–HCl buffer (pH 7.0).

Western blot analysis using the anti-Cx43 primary antibody and horseradish peroxidase (HRP)-conjugated secondary antibody showed expression of Cx43 (43 kDa) in the presence of the enveloped artificial viral capsid (Fig. 2A). The expression of Cx43 in the presence or absence of enveloped capsids was quantified by band density. In the presence of the enveloped capsid consisting of DOTAP/DOPC = 0.2/10, the expression was similar to that of the control (*i.e.* in the absence of the enveloped capsid). However, as the ratio of DOTAP increased, the expression level decreased. These results indicate that the optimal ratio of DOTAP/DOPC for the expression of Cx43 is 0.2/10, and that higher ratios of cationic lipids inhibit expression. It is likely that translation was inhibited by the adsorption of ribosomes or pURE-Cx43 on the excess cationic surface of the enveloped capsid. In addition, the expression level of Cx43 on the enveloped capsid (DOTAP/DOPC = 1/10) increased with increasing plasmid concentration (Fig. 2B). The expression of Cx43 in the presence of β-annulus-EE peptide or DOTAP/DOPC liposome was also observed using western blot analysis; however, no decrease in the expression level of Cx43, caused by the increase of DOTAP, was observed (Fig. S5, ESI†). Probably, the difference in the expression of Cx43 on the enveloped capsid and liposome might be caused by differences in membrane curvature and fluidity.

### FCS analyses of enveloped artificial viral replica embedded with Cx43

**Fig. 3:**
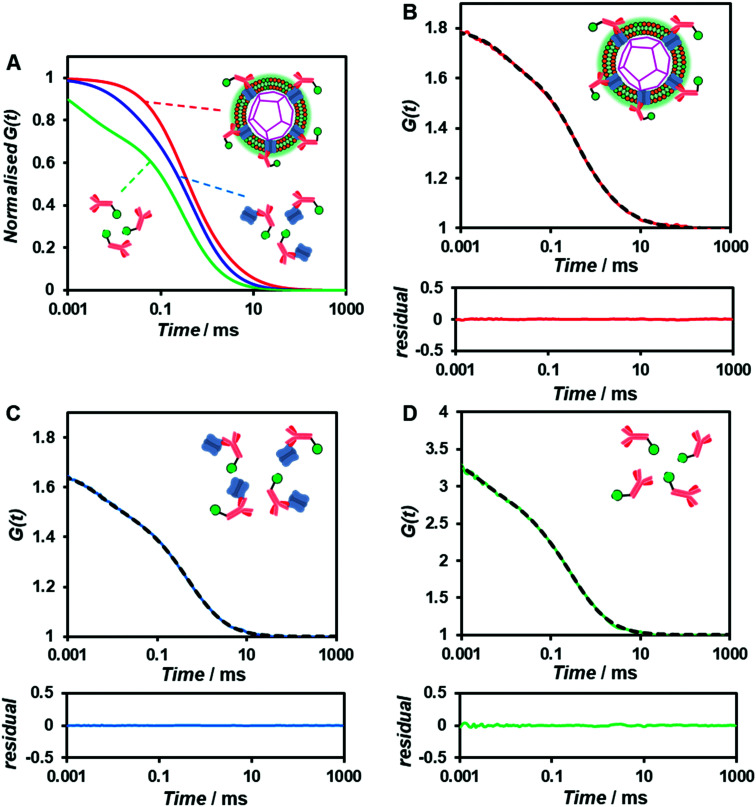
(A) Normalised auto-correlation curves of 10 nM Alexa Fluor 488-labelled anti-Cx43 antibody in the presence of Cx43-embedded viral replica (red), free Cx43 (blue), and in the absence of Cx43 (antibody alone, green) in 10 mM Tris–HCl buffer (pH 7.0) at 25 °C. (B–D) Measured (solid) and fitted (dot) auto-correlation curves of 10 nM Alexa Fluor 488-labelled anti-Cx43 antibody in the presence of (B) Cx43-embedded viral replica ([β-annulus-EE] = 4 μM, [DOTAP] = 12 μM, [DOPC] = 120 μM) or (C) free Cx43, and (D) in the absence of Cx43 (the antibody alone). Cx43 was expressed from 17.3 nM pURE-Cx43 using the PURE system.

To confirm whether the expressed Cx43 is embedded on the surface of the enveloped artificial viral capsid, FCS analysis was conducted by adding the Alexa Fluor 488-labelled anti-Cx43 antibody at 25 °C. FCS analysis can measure spontaneous fluorescence intensity fluctuations in the confocal region and estimate the diffusion time of fluorescent molecules.^54–56^ The normalised auto-correlation function decay obtained from complexes of the Alexa Fluor 488-labelled anti-Cx43 antibody (10 nM) with the Cx43-embedded enveloped artificial viral replica was slower compared with that of the antibody alone and that of the antibody complex with free Cx43 (Fig. 3A). This result indicates the increase in the apparent molecular size of the Alexa Fluor 488-labelled anti-Cx43 antibody resulting from the interaction between it and Cx43 on the enveloped artificial viral replica. The auto-correlation function *G*(*t*) for the mixture of the antibody and enveloped viral replica equipped with Cx43 (Fig. 3B), mixture of the antibody and free Cx43 (Fig. 3C), and the antibody alone (Fig. 3D) were fitted with a dual-component model [Materials and methods, eqn (1)]. Based on the curve-fitting results, we estimated the diffusion time and ratio for the fast and slow components, whereas the apparent hydrodynamic diameter was calculated using the Stokes–Einstein equation [Materials and methods, eqn (3)] as summarised in Table 1. The 83.1% fast component (*τ* = 0.311 ms, *d* = 10.3 nm) obtained from the mixture of the antibody and Cx43-embedded viral replica can be attributed to the free antibody, whereas the 16.9% slow component (*τ* = 3.64 ms, *d* = 120.9 nm) can be attributed to the antibody bound to the Cx43-embedded viral replica alone, or as dimer *via* gap junctions. In contrast, there were no components with an apparent hydrodynamic diameter of approximately 100 nm in FCS measurements of the mixture of the antibody and free Cx43 or antibody alone. The 75.6% slow component (*τ* = 0.538 ms, *d* = 17.8 nm) obtained from the mixture of the antibody and free Cx43 can be attributed to the antibody bound to free Cx43. These results strongly support binding of the Alexa Fluor 488-labelled anti-Cx43 antibody to the Cx43-embedded viral replica. In contrast, FCS analyses of the antibody in the presence of the enveloped capsid without Cx43 showed the presence of only unbound antibody (Fig. S6 and Table S1, ESI†). In addition, FCS analyses of the mixture of antibody and Cx43 expressed in the presence of the β-annulus-EE peptide alone or DOTAP/DOPC liposome did not show any components with an apparent hydrodynamic diameter of 100 nm (Fig. S6 and Table S1, ESI†). Therefore, the enveloped artificial viral capsid may be a suitable platform for embedding membrane proteins.

**Table 1:** Diffusion time (*τ*), ratio (*R*), and apparent diameter (*d*) of Alexa Fluor 488-labelled anti-Cx43 antibody in the presence of Cx43-embedded viral replica ([β-annulus-EE] = 4 μM, [DOTAP] = 12 μM, [DOPC] = 120 μM), free Cx43, and the antibody alone obtained from FCS curve-fitting analyses in 10 mM Tris–HCl buffer (pH 7.0) at 25 °C. Cx43 was expressed from 17.3 nM pURE-Cx43 using the PURE system

	*τ*_1_/ms	*R*_1_/%	*d*_1_/nm	*τ*_2_/ms	*R*_2_/%	*d*_2_/nm
Antibody + Cx43-embedded viral replica	0.311	83.1	10.3	3.64	16.9	120.9
Antibody + free Cx43	0.024	24.4	0.8	0.538	75.6	17.8
Antibody alone	0.002	27.5	0.1	0.310	72.5	10.3

**Fig. 4:**
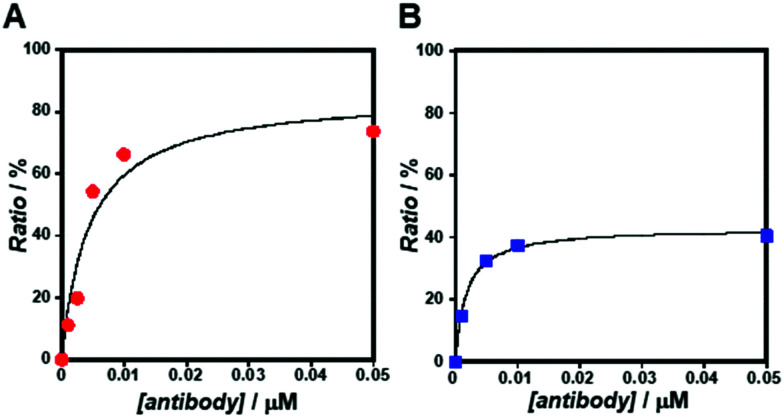
Concentration dependence of Alexa Fluor 488-labelled anti-Cx43 antibody on the ratio of the slow component determined by FCS curve fitting in the presence of (A) free Cx43 and (B) Cx43-embedded viral replica ([β-annulus-EE] = 4 μM, [DOTAP] = 12 μM, [DOPC] = 120 μM) at 25 °C. Cx43 was expressed from 17.3 nM pURE-Cx43 using the PURE system. Binding curve of the antibody to Cx43 is characterised by the Langmuir equation.

As the concentration of the Alexa Fluor 488-labelled anti-Cx43 antibody increased, the ratio of the antibody component binding to free Cx43 (Fig. 4A and Fig. S7, Table S2, ESI†) or Cx43-embedded viral replica (Fig. 4B and Fig. S8, Table S3, ESI†) increased. The binding curves were analysed by the least-squares method using the Langmuir equation [Materials and methods, eqn (4)] to determine the dissociation constants (*K*_d_) and the maximum ratio (*R*_max_). The *K*_d_ and *R*_max_ of the antibody bound to free Cx43 were calculated to be 4.4 ± 1.7 nM and 85.6 ± 10.6, respectively. On the other hand, the *K*_d_ (1.8 ± 0.2 nM) and *R*_max_ (42.9 ± 1.0) of the antibody to Cx43-embedded viral replica were lower compared with those bound to free Cx43. A lower *K*_d_ indicates that the Cx43-embedded viral replica binds more strongly to the antibody than to free Cx43, probably because of the multivalent binding on the viral replica. The lower *R*_max_ of the antibody to Cx43-embedded viral replica may be caused by different directions of Cx43 embedded to the enveloped capsids. That is, the antibody can bind to the C-terminal of Cx43 exposed outside of the envelope, but not to the C-terminal shielded inside.

### TEM observation of the binding of the antibody to the enveloped artificial viral replica embedded with a membrane protein

**Fig. 5:**
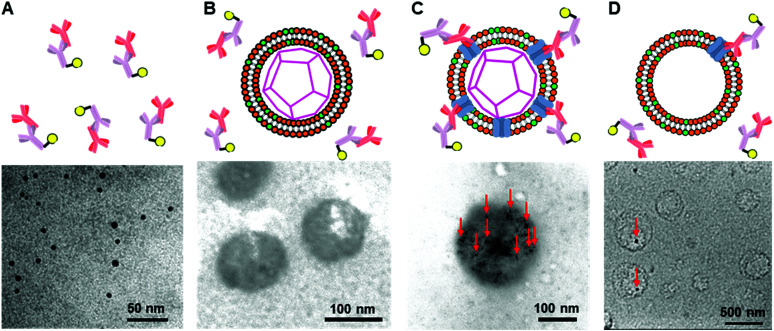
TEM images of (A) anti-Cx43 antibody (42 nM) and the gold nanoparticle-labelled secondary antibody (14.8 nM) against anti-Cx43 antibody, the mixture of the anti-Cx43 antibody and the gold nanoparticle-labelled secondary antibody with (B) the enveloped viral capsid ([β-annulus-EE] = 4 μM, [DOTAP] = 12 μM, [DOPC] = 120 μM), (C) Cx43-embedded viral replica ((DOTAP/DOPC = 1/10) [β-annulus-EE] = 4 μM, [DOTAP] = 12 μM, [DOPC] = 120 μM), and (D) Cx43-expressed liposome ([DOTAP] = 12 μM, [DOPC] = 120 μM) in 10 mM Tris–HCl buffer (pH 7.0). Cx43 was expressed from 17.3 nM pURE-Cx43 using PURE system. TEM samples were stained with EM stainer. Red arrows indicate the gold nanoparticle-labelled secondary antibody.

To further confirm the presence of Cx43 on the enveloped capsid, we directly observed TEM images of the Cx43-embedded viral replica with the anti-Cx43 primary antibody and gold nanoparticle-labelled secondary antibody (Fig. 5). TEM images of the complex of the anti-Cx43 primary antibody and gold nanoparticle-labelled secondary antibody as a control showed dot-like structures of approximately 5 nm (Fig. 5A). The enveloped artificial viral capsid without Cx43 did not show the presence of gold nanoparticles on the surface of the spherical structures, even with the mixing of the gold nanoparticle-labelled secondary antibody (Fig. 5B). In contrast, TEM images of the Cx43-embedded viral replica (expressed from 17.3 nM pURE-Cx43) showed many dot-like structures on the surface of the spherical structures (Fig. 5C). This indicates that Cx43 is embedded on the enveloped artificial viral replica. TEM images of Cx43-expressing liposomes consisting of DOTAP/DOPC at a 1/10 ratio showed the presence of a few dot-like structures per liposome (Fig. 5D).

**Fig. 6:**
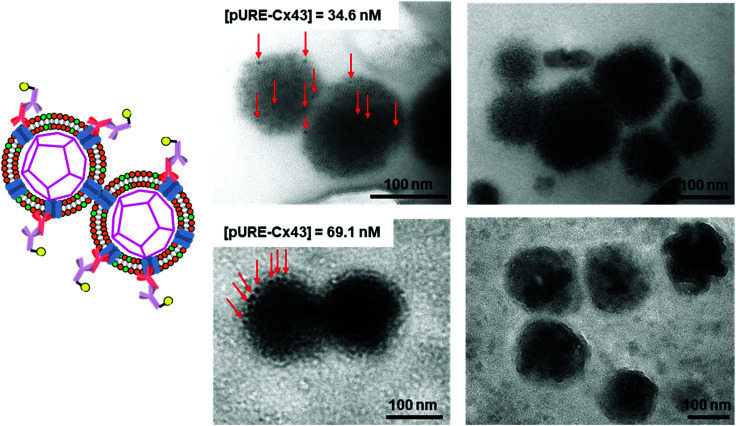
TEM images of anti-Cx43 antibody (42 nM) and the gold nanoparticle-labelled secondary antibody (14.8 nM) against anti-Cx43 antibody under the Cx43-embedded viral replica ((DOTAP/DOPC = 1/10) [β-annulus-EE] = 4 μM, [DOTAP] = 12 μM, [DOPC] = 120 μM) in 10 mM Tris–HCl buffer (pH 7.0). Cx43 was expressed from 34.6 or 69.1 nM pURE-Cx43 using PURE system. TEM samples were stained with EM stainer. Red arrows indicate the gold nanoparticle-labelled secondary antibody.

Next, we increased the concentration of the plasmid used for expressing Cx43 ([pURE-Cx43] = 34.6, 69.1 nM) and observed TEM images using the same method. Interestingly, spherical structures connected to each other were abundant (Fig. 6). These images strongly support the formation of gap junction structures among Cx43-embedded viral replicas. In addition, as the plasmid concentration increased, the density of the gold nanoparticle-labelled secondary antibody bound to the Cx43-embedded viral replica surface appeared to increase, suggesting the increase in the amount of Cx43 embedded on the envelope (Fig. S9, ESI†). Compared to the TEM images without the antibody (Fig. 1B and Fig. S4, ESI†), the aggregation of spherical structures was minimally observed. These results suggest that the antibodies bound to Cx43 on the envelope inhibit the formation of excessive gap junctions between Cx43-embedded viral replica. The average distance between the gap junctions of the spherical structures was estimated to be approximately 20 nm, based on the TEM images. This suggests that the enveloped artificial viral replicas are tightly adherent to one another (Fig. S10, ESI†). The particle size distribution from the TEM images of the Cx43-embedded viral replica at each plasmid concentration was 140 ± 76 nm at 17.3 nM, 143 ± 38 nm at 34.6 nM, and 165 ± 25 nm at 69.1 nM (Fig. S11–13, ESI†). These particle sizes were larger than those estimated from the FCS (Table 1), and those observed in the TEM image without antibody (Fig. 1B). The increase in particle size may be caused by the binding of primary and secondary antibodies to the Cx43-embedded viral replica.

### Transport of 5-TMR from the Cx43-embedded viral replica to HepG2 cells

**Fig. 7:**
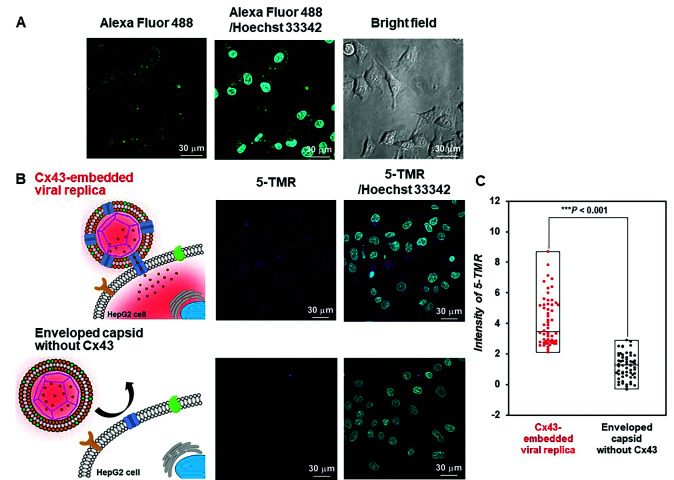
Transport of 5-TMR from Cx43-embedded viral replica into HepG2 cells. (A) CLSM images of HepG2 cells incubated with 5 μM Alexa Fluor 488-labelled anti-Cx43 antibody. Channels for Alexa Fluor 488 (green), Hoechst 33342 (cyan), and bright field for CLSM images. (B) CLSM images of HepG2 cells incubated with 5-TMR-encapsulated Cx43-embedded viral replica or enveloped capsid without Cx43 ([β-annulus-EE] = 10 μM, [DOTAP] = 30 μM, [DOPC] = 300 μM, [5-TMR] = 1.7 μM). Cx43 was expressed from 17.3 nM pURE-Cx43 using PURE system. Channels for 5-TMR (magenta) and Hoechst 33342 (cyan) for CLSM images. (C) Box plot of the fluorescence intensity distribution of 5-TMR in HepG2 cells normalised outside of cells (*N* = 60). The *P*-value was calculated by one-way analysis of variance followed by Mann–Whitney *U* test.

Evaluation of the functionality of the Cx43-embedded viral replica is important to show that a functional Cx43 is properly embedded on the envelope. Therefore, we performed the transport experiments of fluorescent small dyes into Cx43-expressing cells through the formation of gap junctions between Cx43-embedded viral replicas and Cx43-expressing cells. A 5-carboxytetramethylrhodamine (5-TMR)-encapsulated artificial viral capsid was prepared by adding a solution of 5-TMR in DMSO to β-annulus-EE powder, then dissolving it in 10 mM Tris–HCl buffer (pH 7.0). The capsid was then complexed with DOTAP/DOPC to construct a 5-TMR-encapsulated enveloped capsid. The particle size of the 5-TMR-encapsulated capsid obtained from DLS was 30 ± 12 nm (Fig. S14A, ESI†), whereas that of the 5-TMR-encapsulated enveloped capsid was 47 ± 15 nm (Fig. S14B, ESI†), indicating that particle size was increased by complexation. After removing free 5-TMR by dialysis, the concentration of 5-TMR encapsulated in enveloped capsid was determined by UV/vis spectroscopy. Cx43 was embedded into the 5-TMR-encapsulated enveloped viral replica by PURE system using 17.3 nM pURE-Cx43. We used HepG2 cells (human hepatoma cell line) that are known to express Cx43.^57^ The expression of Cx43 was confirmed by binding of the Alexa Fluor 488-labelled anti-Cx43 antibody to the cell surface in the CLSM image (Fig. 7A). After addition of the 5-TMR-encapsulated Cx43-embedded viral replica or non-embedded viral replica to HepG2 cells and further incubation for 1 h, the transport activity of 5-TMR was evaluated by CLSM. As a result, 5-TMR-derived fluorescence was observed inside the cells by adding the Cx43-embedded viral replica, while the fluorescence was minimally observed by adding the enveloped capsid without Cx43 (Fig. 7B). The fluorescence intensity distribution of the Cx43-embedded viral replica showed a significant difference compared with the enveloped capsid without Cx43 (Fig. 7C, S15, and S16, *P* < 0.001). These results indicate that 5-TMR could be transported from the Cx43-embedded viral replica into Cx43-expressing cells through the gap junctions.

## Conclusions

We successfully constructed a membrane protein Cx43-embedded viral replica using a cell-free protein expression system (PURE system). Western blot analysis revealed that Cx43 was well expressed in the presence of the enveloped artificial viral capsid. FCS analyses confirmed the presence of a component of the anti-Cx43 antibody bound to the Cx43 embedded on the envelope surface. TEM images obtained using the anti-Cx43 antibody with a gold nanoparticle-labelled secondary antibody strongly support the existence of many Cx43 molecules embedded on the envelope. Interestingly, as the plasmid concentration increased, gap junction structures between Cx43 were observed by TEM. In addition, we succeeded in selectively transporting fluorescent dyes from the Cx43-embedded viral replica into Cx43-expressing cells, probably through gap junctions. These results demonstrated that functional membrane proteins were embedded on the lipid bilayer surface of artificial enveloped capsids. In the future, drug transport using Cx43-embedded viral replicas into Cx43-expressing cells will be the next challenge. Our strategy provides a new concept for constructing multimolecular crowding complexes equipped with cellular proteins.

## Materials and methods

### General

Reversed-phase HPLC was performed at ambient temperature using a Shimadzu LC-6AD liquid chromatography system equipped with a UV/vis detector (220 nm, Shimadzu SPD-10AVvp) and an Inertsil WP300 C18 column (250 × 4.6 mm and 250 × 20 mm, GL Science). MALDI-TOF mass spectra were obtained using an Autoflex-T2 instrument (Bruker Daltonics) in linear/positive mode with a-cyano-4-hydroxy cinnamic acid (α-CHCA) as a matrix. Ultrapure water with high resistivity (>18 MΩ cm) was purified using a Millipore Purification System (Milli-Q water), and was used as a solvent for the peptides. Reagents were obtained from a commercial source and used without further purification.

### Synthesis of β-annulus-EE peptide

The peptide H-Ile-Asn(Trt)-His(Trt)-Val-Gly-Gly-Thr(*t*Bu)-Gly-Gly-Ala-Ile-Met-Ala-Pro-Val-Ala-Val-Thr(*t*Bu)-Arg(Pbf)-Gln(Trt)-Leu-Val-Gly-Ser(*t*Bu)-Glu(O*t*Bu)-Glu(O*t*Bu)-Alko-PEG resin was synthesised on Fmoc-Glu(O*t*Bu)-Alko-PEG resin (494 mg, 0.123 mmol g^−1^; Watanabe Chemical Ind. Ltd) using Fmoc-based coupling reactions (4 equiv. of Fmoc amino acid). *N*-Methylpyrrolidone (NMP) solution containing (1-cyano-2-ethoxy-2-oxoethylidenaminooxy) dimethylamino-morpholino-carbenium hexafluorophosphate (4 equiv. of Fmoc amino acid) and diisopropylethylamine (4 equiv. of Fmoc amino acid) used as the coupling reagent. Fmoc deprotection was achieved using 20% piperidine in *N*,*N*-dimethylformamide. Progression of the coupling reaction and Fmoc deprotection was monitored by TNBS and a chloranil test kit (Tokyo Chemical Industry Co., Ltd). Peptidyl resins were washed with NMP and were then dried under vacuum. The peptide was deprotected and cleaved from the resin by treatment with a cocktail of trifluoroacetic acid (TFA)/1,2-ethanedithiol/triisopropylsilane/water = 3.76/0.1/0.04/0.1 (mL) at room temperature for 4 h. The reaction mixture was filtered to remove resins, and the filtrate was concentrated *in vacuo*. The peptide was precipitated by adding methyl *tert*-butyl ether (MTBE) to the residue, and the supernatant was decanted. After being washed three times with MTBE, the crude peptide was lyophilised and purified using reverse-phase HPLC with elution of a linear gradient of CH_3_CN/water containing 0.1% TFA (5/95 to 100/0 over 100 min). The fraction containing the desired peptide was lyophilised to yield 33.5 mg of a flocculent solid (35% yield). MALDI-TOF MS (matrix: α-CHCA): *m*/*z* = 2563 [M]^+^.

### Complexation of the β-annulus-EE peptide and DOTAP/DOPC lipids

Stock solutions of DOTAP (Avanti Polar Lipids, 10 mM, 3 μL) and DOPC (Tokyo Chemical Industry Co., Ltd, 10 mM, 30 μL) in chloroform, methanol (33 μL) and chloroform (66 μL) were combined in a glass tube and dried *in vacuo* for 6 h. The resulting lipid film was hydrated with a solution (50 μM, 200 μL) of β-annulus-EE peptide in 10 mM Tris–HCl buffer (pH 7.0) at 50 °C for 1 h, in which the cation/anion charge ratio of the complex was kept at 1 : 1.

### Cell-free synthesis of Cx43 on the enveloped viral capsid

PURE frex2.1 (GeneFrontier Co., Ltd) was used for cell-free protein synthesis. The Cx43 plasmid (pURE-Cx43) was constructed based on methods described in a published report.^58^ A plasmid fragment covering the entire coding region of rat Cx43^59^ was amplified from a rat heart cDNA library by polymerase chain reaction with the following primers: For 5′-TAAGTGAAAGAGAGGTGCCC-3′ (178 to 197 bp); Rev 5′-CTCCTCCATAATCGACAGCT-3′ (1386 to 1367 bp). Aliquots of aqueous solutions (1 μg μL^−1^) of the plasmid and the enveloped artificial viral capsid (8 μL) in 10 mM Tris–HCl buffer (pH 7.0) were added to the reaction mixture of solution I (12 μL, 11 μL, or 9 μL), solution II (1 μL), solution III (1 μL), cysteine solution (1 μL), and the glutathione solution (1 μL) included in PURE frex 2.1. The final concentration of the Cx43 plasmid was maintained at 17.3 nM, 34.6 nM, and 69.1 nM by various volumes of plasmid solution and solution I. The samples were incubated for 4 h at 37 °C. Following protein synthesis, the samples were stored at 4 °C or used immediately.

### DLS

The DLS of the Cx43-embedded viral replica (expressed from 17.3 nM pURE-Cx43) in 10 mM Tris–HCl buffer (pH 7.0) was measured at 25 °C using Zetasizer Nano ZS (MALVERN) with an incident He–Ne laser (633 nm) and ZEN2112-Low volume glass cuvette cell. During measurements, count rates (sample scattering intensities) were also recorded. Correlation times of the scattered light intensities *G*(*τ*) were measured several times and their means were calculated for the diffusion coefficient. Hydrodynamic diameters of scattering particles were calculated using the Stokes–Einstein equation.

### TEM

A solution (42 nM, 5 μL) of the enveloped viral replica embedded with Cx43 in 10 mM Tris–HCl buffer (pH 7.0) and the mouse IgG anti-Cx43 monoclonal antibody (BD Transduction) in the same buffer were mixed and incubated overnight at 25 °C. An aqueous glycerol solution of gold nanoparticle-labelled goat anti-mouse IgG secondary antibody (Sigma-Aldrich, 14.8 nM, 10 μL) was then added to the mixture and was incubated for 3 h at 25 °C. Aliquots (5 μL) of the sample aqueous solutions were applied to the hydrophilised carbon-coated Cu-grids (C-SMART Hydrophilic TEM grids, ALLANCE Biosystems) for 1 min, and were then removed. Subsequently, the TEM grids were immersed in the staining solution (5 μL): 25% EM stainer (Nisshin EM Co., Ltd) for 10 min, and were then removed. After the sample-loaded carbon-coated grids were dried *in vacuo*, they were observed using TEM (JEOL JEM 1400 Plus) at an accelerating voltage of 80 kV.

### Western blot analysis

The enveloped viral replica samples embedded with Cx43 in 10 mM Tris–HCl buffer (pH 7.0) were ultracentrifuged (at 25 000 rpm, for 2 min) to remove free liposomes, and were diluted twice with sample buffer solution with reducing reagent (6×) for SDS-PAGE (375 mM Tris/HCl, pH 6.8, 0.03% bromophenol blue, glycerol, anionic surfactant, reducing agent, Nacalai Tesque Inc.). After incubation at 95 °C for 5 min, the samples were separated using SDS/PAGE (gel concentration of 15%, 20 mA, 300 V, 85 min). For western blot analysis, the gel-bound protein bands were electrotransferred to the poly(vinylidene difluoride) membrane. The membrane was blocked by incubating for 60 min at 4 °C in 20% Blocking One (30 mL, Nacalai Tesque) in 1× Tris Buffered Saline (TBS, 5 mM Tris, 13.8 mM NaCl 0.27 mM KCl, pH 7.4) containing 0.1% (v/v) Tween-20 (TBS-T). The blocked membrane was then incubated overnight at 4 °C in TBS-T and mouse IgG anti-Cx43 monoclonal antibody [0.1% dilution (v/v) with 10% Blocking One in TBS-T, 0.42 nM]. The membrane was washed with TBS-T four times, and was subsequently incubated for 60 min at 4 °C with HRP-conjugated goat anti-mouse IgG [Cosmo Bio Co., Ltd; 0.1% dilution (v/v) with 10% Blocking One in TBS-T] for the anti-Cx43 monoclonal antibody. After being washed with TBS-T four times, blots were developed using a chemiluminescence method (ECL; GE Healthcare). The band images were obtained with a LAS-4000EPUVmini (Fujifilm Co., Ltd) with an exposure time of 30 min.

### FCS

The Alexa Fluor 488-labelled anti-Cx43 antibody (Bioss Inc.) was added to the aqueous solution of the enveloped viral replica embedded with Cx43 (the final concentration of the antibody was 10 nM), and was then incubated at 25 °C for 1 h. FCS analyses of the solutions were conducted on an FCS compact BL/GR (Hamamatsu Photonics Co., Ltd) in a microwell slide (25 μL) using a 473-nm laser (75 μW) at 25 °C. Each measurement was recorded for 150 s (period of a single measurement: 5 s; looptime: 30 times). The diffusion time (*τ*) and ratio (*R*) of the fast (*τ*_1_, *R*_1_) and slow (*τ*_2_, *R*_2_) components were obtained by curve-fitting the auto-correlation function *G*(*t*) obtained from FCS measurements based on (eqn (1))^60^1
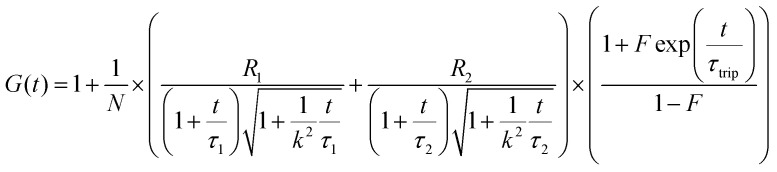
where *τ* is the diffusion time of the Alexa Fluor 488-labelled anti-Cx43 antibody in the detection area, *N* is the average number of Alexa Fluor 488-labelled anti-Cx43 antibodies in the detection area, *k* is the structural parameter, and *F* is triplet component ratio. The hydrodynamic radiuses (*r*_1_, *r*_2_) of the Alexa Fluor 488-labelled anti-Cx43 antibody were calculated using eqn (2) and (3). The hydrodynamic diameters (*d*_1_, *d*_2_) were calculated by doubling *r*_1_ and *r*_2_:2
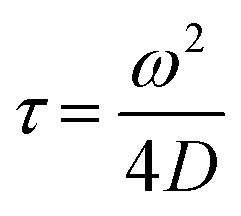
3
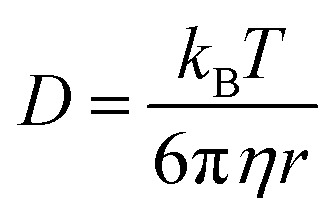
where *ω* is the radius of the detection area, *T* is the absolute temperature, *k*_B_ is the Boltzmann constant, *η* is the viscosity of the solvent, and *D* is the diffusion coefficient of the Alexa Fluor 488-labelled antibody. The value of *ω* was evaluated through reference measurement using Alexa 488 (*D* = 414 μm^2^ s^−1^).^61^ The diffusion time of Alexa 488 was 0.0356 ms.

To calculate the dissociation constant *K*_d_ for the complex of Cx43 and Alexa Fluor 488-labelled anti-Cx43 antibody, the following equation (Langmuir's equation) was used.4
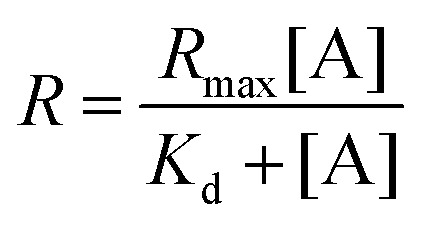
where [A] is the antibody concentration, *R* is the ratio of antibody bound to Cx43 at the antibody concentration, and *R*_max_ is the maximum ratio of antibody bound to Cx43.

### Encapsulation of 5-TMR into the Cx43-embedded viral replica

β-Annulus-EE peptide encapsulated 5-TMR was prepared by adding 5-TMR (Ambeed, Inc.) stock solution in DMSO (10 mM, 1 μL) to the β-annulus-EE powder, and then vortexing after adding 10 mM Tris–HCl buffer (199 μL). Stock solutions of DOTAP (10 mM, 3 μL) and DOPC (10 mM, 30 μL) in chloroform, methanol (33 μL) and chloroform (66 μL) were combined in a glass tube and dried *in vacuo* for 6 h. The resulting lipid film was hydrated with a solution of 5-TMR-encapsulated artificial viral capsid ([β-annulus-EE] = 156.3 μM, [5-TMR] = 26.6 μM, 64 μL) in 10 mM Tris–HCl buffer (pH 7.0) at 50 °C for 1 h. Free liposomes were removed from the complex by ultracentrifugation at 25 000 rpm for 2 min, using an Optima MAX-TL Ultracentrifuge (Beckman Coulter, Inc.), and free 5-TMR was removed by dialysis (1 kDa cut off). The solution containing the 5-TMR-encapsulated enveloped capsid in 10 mM Tris–HCl buffer (pH 7.0, 8 μL) and aqueous solution aliquots (1 μg μL^−1^, 1 μL) of the Cx43 plasmid were added to the reaction mixture of solution I (12 μL), solution II (1 μL), solution III (1 μL), cysteine solution (1 μL), and the glutathione solution (1 μL) included in PURE frex 2.1. The sample was incubated for 4 h at 37 °C. The concentration of 5-TMR encapsulated in the enveloped capsid was determined by UV/vis spectroscopy (NanoDrop One, Thermo Scientific) as 8.5 μM.

### Transport of 5-TMR from the Cx43-embedded viral replica to HepG2 cells

HepG2 cells (RIKEN BioResource Research Center, Japan) were cultured in Dulbecco's modified Eagle's medium (DMEM). The medium contained 10% fetal bovine serum (FBS, v/v), 100 μg mL^−1^ streptomycin, 100 units per mL penicillin, 1 mM sodium pyruvate, and 1% MEM nonessential amino acids (v/v, Sigma M7145). Cells were maintained at 37 °C in a 5% CO_2_ incubator, and a subculture was performed every 3–4 days.

HepG2 cells were seeded onto a single-well glass bottom dish at 2.0 × 10^4^ cells per well in a final volume of 100 μL, and were incubated for 24 h at 37 °C, 5% CO_2_. Solutions of the Alexa Fluor 488-labelled anti-Cx43 antibody (5 μM, 60 μL) in DMEM (+10% FBS), the Cx43-embedded viral replica, or enveloped viral capsid without Cx43 (50 μL) were added to the cells and these were incubated for 1 h, at 37 °C, 5% CO_2_. After removal of the solution, 10 μg mL^−1^ Hoechst 33342 (80 μL) was added to the cells and another incubation phase was carried out for 10 min at 37 °C, 5% CO_2_. After being washed with PBS, the medium was added to the cells and CLSM was performed. Alexa Fluor 488 was excited at 499 nm and was observed through a 520 nm emission band-pass filter (green). 5-TMR was excited with 553 nm and was observed through a 577 nm emission band-pass filter (magenta). Hoechst 33342 was excited with 352 nm and was observed through a 455 nm emission band-pass filter (cyan). The fluorescence intensity of 5-TMR transported into the HepG2 cells was measured from the fluorescence images by subtracting the background intensity using Image J software (*N* = 60).

## Conflicts of interest

There are no conflicts of interest to declare.

## Supplementary Material

CB-003-D1CB00166C-s001
