# Supplementary material for: Embedding a membrane protein into an enveloped artificial viral replica
Source: RSC Chem Biol. 2021 Dec 21;3(2):231–41. doi: 10.1039/d1cb00166c (PMC8827153; doi:10.1039/d1cb00166c)
Supplement: CB-003-D1CB00166C-s001 [file CB-003-D1CB00166C-s001.pdf]

Electronic Supplementary Material (ESI) for RSC Chemical Biology.
This journal is © The Royal Society of Chemistry 2021

Electronic Supplementary Information

Embedding Membrane protein into Enveloped Artificial Viral Replica

Hiroto Furukawa,[a] Hiroshi Inaba,[ab] Yoshihiro Sasaki,[c] Kazunari Akiyoshi,[c] and Kazunori Matsuura*[ab]

[a] Department of Chemistry and Biotechnology, Graduate School of Engineering, Tottori University, Koyama-Minami 4-101, Tottori 680-8552, Japan

[b] Centre for Research on Green Sustainable Chemistry, Tottori University, Koyama-Minami 4-101, Tottori 680-8552, Japan

[c] Department of Polymer Chemistry, Graduate School of Engineering, Kyoto University, Katsura, Nishikyo-ku,Kyoto 615-8510, Japan

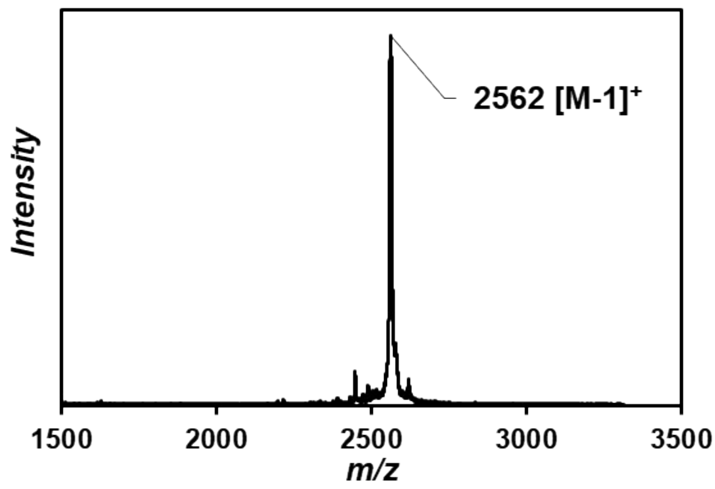

Figure S1. MALDI-TOF-MS of purified *β*-annulus-EE peptide.

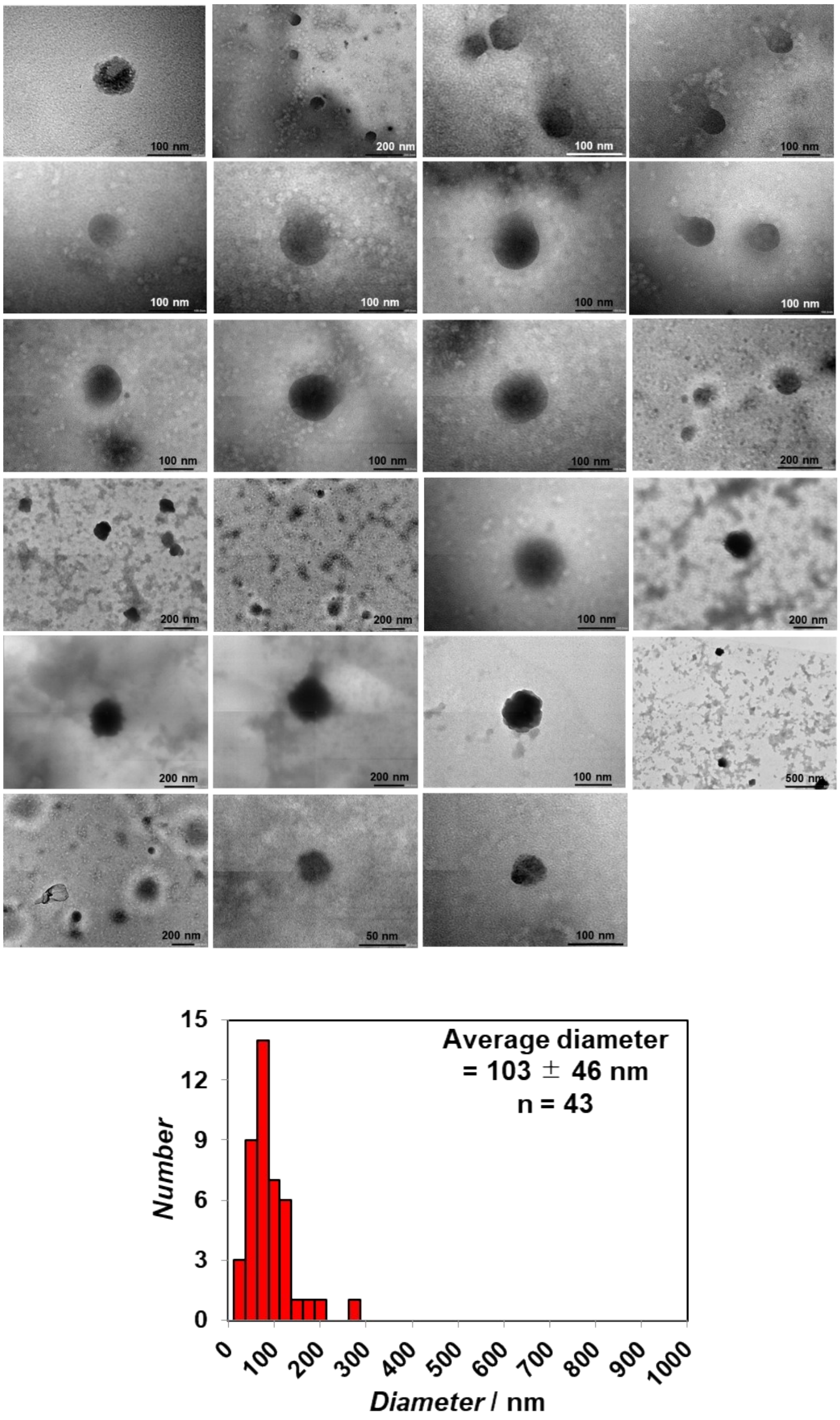

Figure S2. TEM images of Cx43-embedded viral replica ([β-annulus-EE] = 16 μM, [DOTAP] = 48 μM, [DOPC] = 480 μM) and size distribution. Cx43 was expressed from 17.3 nM pURE-Cx43 using PURE system. The sample was stained with EM stainer.

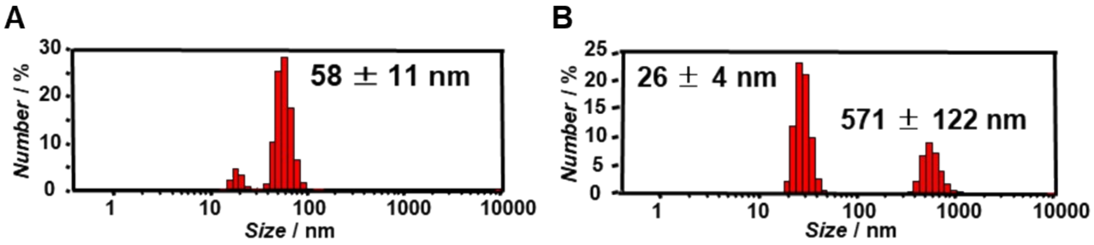

Figure S3. Size distributions obtained from DLS of Cx43-embedded viral replica ([*β*-annulus-EE] = 4 μM, [DOTAP] = 12 μM, [DOPC] = 120 μM). Cx43 was expressed from (A) 17.3, (B) 34.6 nM pURE-Cx43 using PURE system.

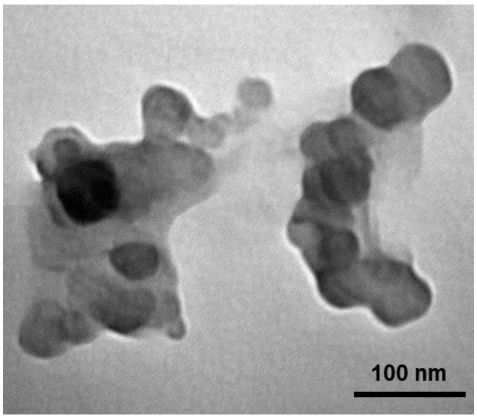

Figure S4. TEM image of Cx43-embedded viral replica ([*β*-annulus-EE] = 16 μM, [DOTAP] = 48 μM, [DOPC] = 480 μM). Cx43 was expressed from 69.1 nM pURE-Cx43 using PURE system. The sample was stained with EM stainer.

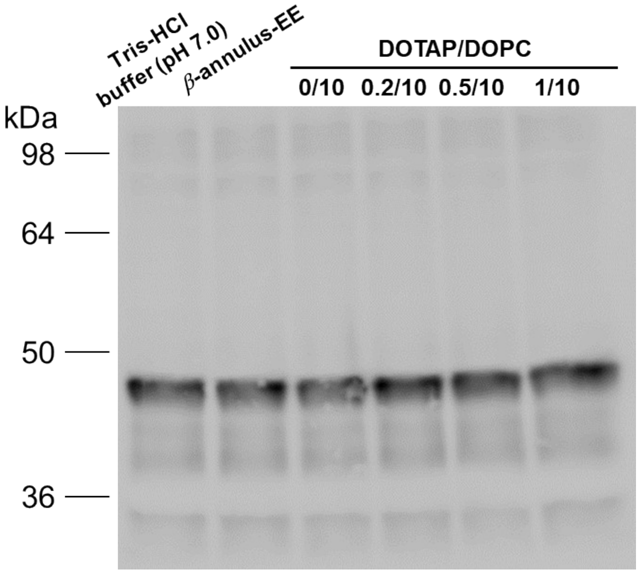

Figure S5. Western blot analysis of Cx43 expressed in the presence of 16 μM β-annulus-EE or DOTAP/DOPC liposome ([DOPC] = 480 μM (DOTAP/DOPC = 0/10); [DOTAP] = 9.6 μM, [DOPC] = 480 μM (DOTAP/DOPC = 0.2/10); [DOTAP] = 24 μM, [DOPC] = 480 μM (DOTAP/DOPC = 0.5/10); [DOTAP] = 48 μM, [DOPC] = 480 μM (DOTAP/DOPC = 1/10)). Cx43 was expressed from 17.3 nM pURE-Cx43 using PURE system.

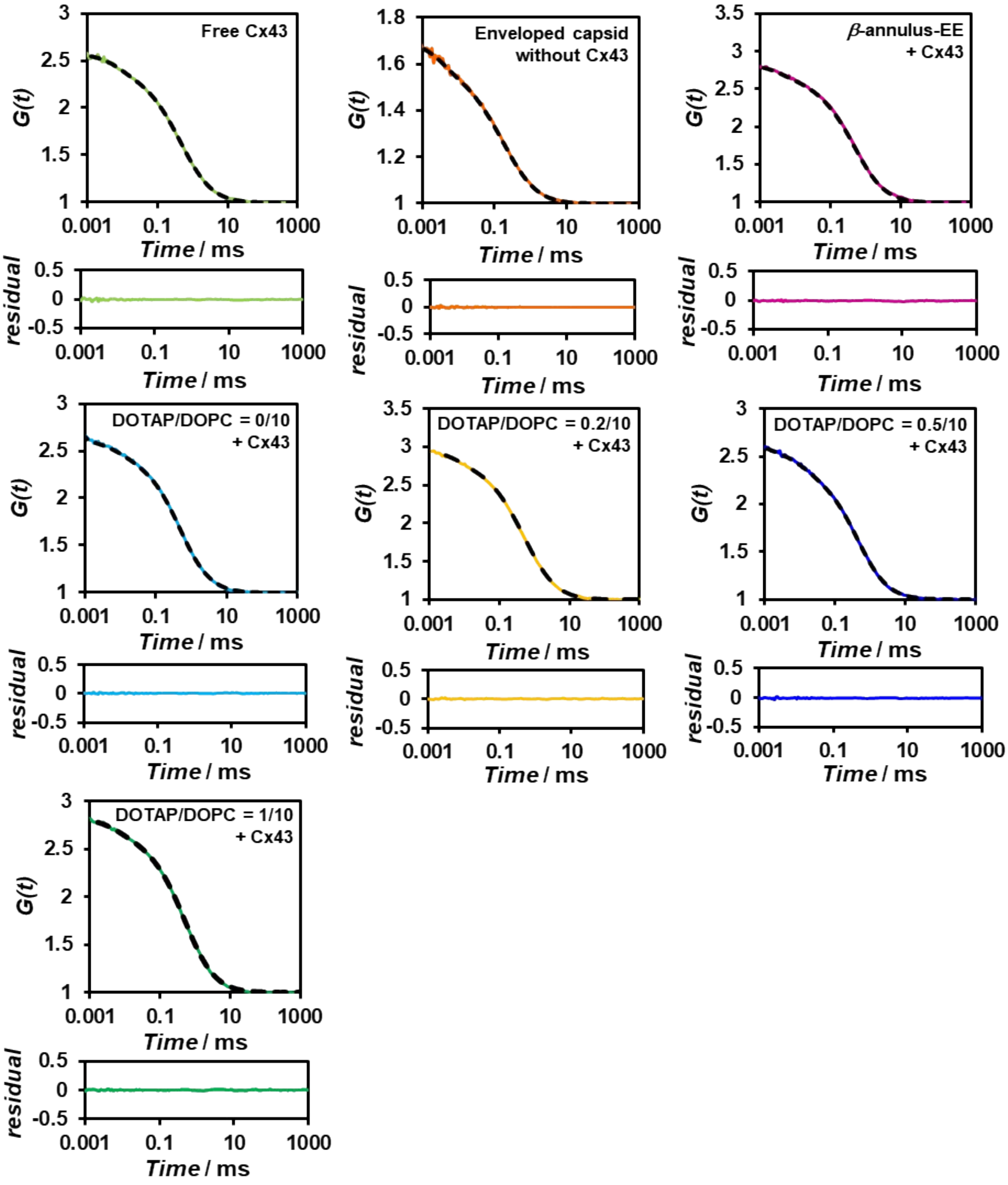

Figure S6. FCS analyses of Alexa Fluor 488-labelled anti-Cx43 antibody (10 nM) in the presence of free Cx43, enveloped capsid without Cx43 ([*β*-annulus-EE] = 4 μM, [DOTAP] = 12 μM, [DOPC] = 120 μM), 4 μM *β*-annulus-EE + Cx43, or DOTAP/DOPC liposomes + Cx43 ([DOPC] = 120 μM (DOTAP/DOPC = 0/10); [DOTAP] = 2.4 μM, [DOPC] = 120 μM (DOTAP/DOPC = 0.2/10); [DOTAP] = 6 μM, [DOPC] = 120 μM (DOTAP/DOPC = 0.5/10); [DOTAP] = 12 μM, [DOPC] = 120 μM (DOTAP/DOPC = 1/10)) in 10 mM Tris-HCl buffer (pH 7.0) at 25°C. Cx43 was expressed from 17.3 nM pURE-Cx43 using PURE system.

Table S1. Diffusion time (τ), ratio (R), and apparent diameter (d) of Alexa Fluor 488-labelled anti-Cx43 antibody (10 nM) in the presence of free Cx43, enveloped capsid without Cx43 ([β-annulus-EE] = 4 μM, [DOTAP] = 12 μM, [DOPC] = 120 μM), 4 μM β-annulus-EE + Cx43, or DOTAP/DOPC liposomes + Cx43 ([DOPC] = 120 μM (DOTAP/DOPC = 0/10); [DOTAP] = 2.4 μM, [DOPC] = 120 μM (DOTAP/DOPC = 0.2/10); [DOTAP] = 6 μM, [DOPC] = 120 μM (DOTAP/DOPC = 0.5/10); [DOTAP] = 12 μM, [DOPC] = 120 μM (DOTAP/DOPC = 1/10)) obtained from FCS curves at 10 mM Tris-HCl buffer (pH 7.0) at 25°C. Cx43 was expressed from 17.3 nM pURE-Cx43 using PURE system.

	τ_1 / ms	R_1 / %	d_1 / nm	τ_2 / ms	R_2 / %	d_2 / nm
Antibody + Free Cx43	0.0835	24.8	2.7	0.593	75.2	19.2
Antibody + enveloped capsid without Cx43	0.0371	34.7	1.2	0.275	65.3	9.1
Antibody + β-annulus-EE + Cx43	0.0362	17.4	1.2	0.538	82.6	17.4
Antibody + DOTAP/DOPC liposome DOTAP/DOPC = 0/10 + Cx43	0.0111	16.9	0.4	0.497	83.1	16.1
Antibody + DOTAP/DOPC liposome DOTAP/DOPC = 0.2/10 + Cx43	0.0994	26.7	3.2	0.690	73.3	22.3
Antibody + DOTAP/DOPC liposome DOTAP/DOPC = 0.5/10 + Cx43	0.0140	22.8	0.5	0.461	77.2	14.9
Antibody + DOTAP/DOPC liposome DOTAP/DOPC = 1/10 + Cx43	0.0321	14.7	1.0	0.534	85.3	17.3

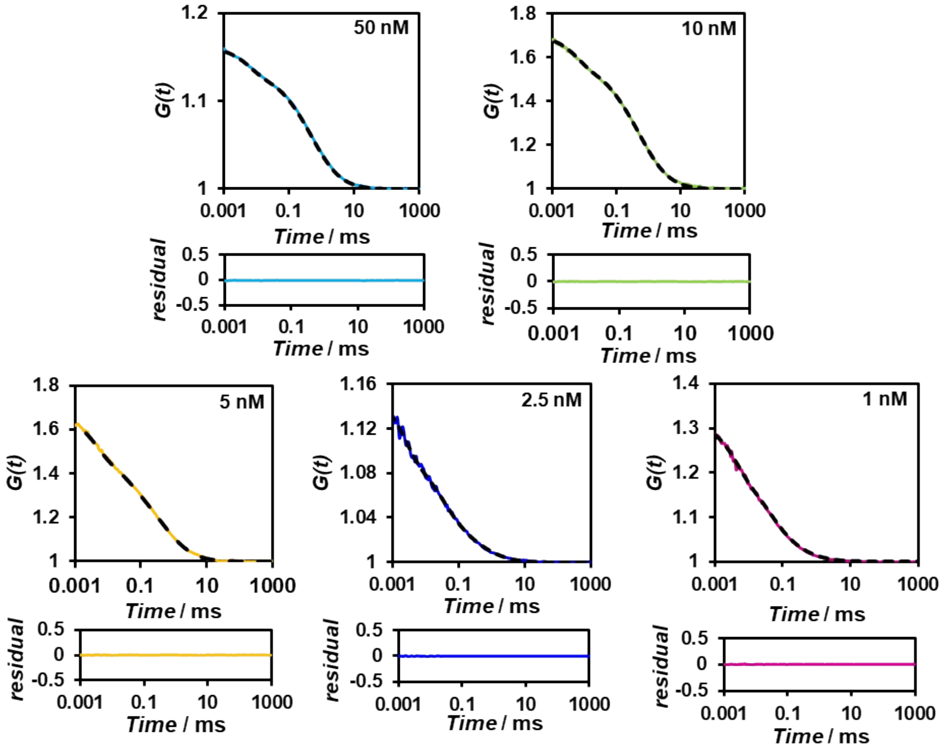

Figure S7. FCS analyses of Alexa Fluor 488-labelled anti-Cx43 antibody (1, 2.5, 5, 10, 50 nM) in the presence of free Cx43 in 10 mM Tris-HCl buffer (pH 7.0) at 25°C. Cx43 was expressed from 17.3 nM pURE-Cx43 using PURE system.

Table S2. Diffusion time (τ), ratio (R), and apparent diameter (d) of Alexa Fluor 488-labelled anti-Cx43 antibody (1-50 nM) in the presence of free Cx43 obtained from FCS curves at 10 mM Tris-HCl buffer (pH 7.0) at 25°C. Cx43 was expressed from 17.3 nM pURE-Cx43 using PURE system.

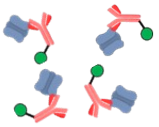

	τ_1 / ms	R_1 / %	d_1 / nm	τ_2 / ms	R_2 / %	d_2 / nm
1 nM	0.0411	89.0	1.5	0.623	11.0	22.7
2.5 nM	0.034	80.2	1.2	0.757	19.8	25.7
5 nM	0.048	45.7	1.7	0.656	54.3	23.9
10 nM	0.097	34.0	3.5	0.784	66.1	28.5
50 nM	0.106	26.3	3.9	0.666	73.7	24.2

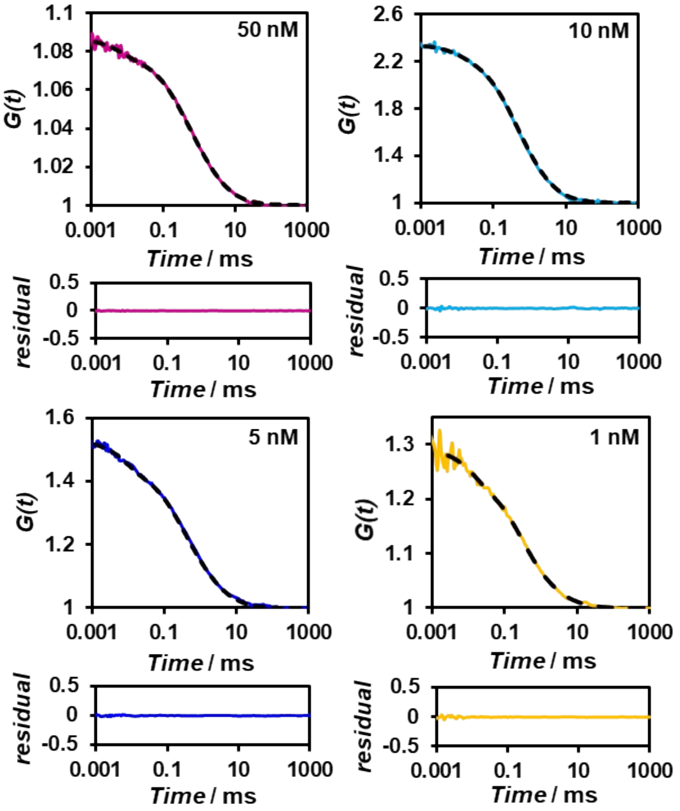

Figure S8. FCS analyses of Alexa Fluor 488-labelled anti-Cx43 antibody (1, 5, 10, 50 nM) in the presence of Cx43-embedded viral replica ([β-annulus-EE] = 4 μM, [DOTAP] = 12 μM, [DOPC] = 120 μM (DOTAP/DOPC = 1/10)) in 10 mM Tris-HCl buffer (pH 7.0) at 25°C. Cx43 was expressed from 17.3 nM pURE-Cx43 using PURE system.

Table S3. Diffusion time (τ), ratio (R), and apparent diameter (d) of Alexa Fluor 488-labelled anti-Cx43 antibody (1, 5, 10, 50 nM) in the presence of Cx43-embedded viral replica obtained from FCS curves at 10 mM Tris-HCl buffer (pH 7.0) at 25°C. Cx43 was expressed from 17.3 nM pURE-Cx43 using PURE system.

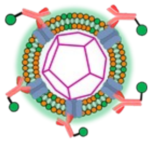

	τ_1 / ms	R_1 / %	d_1 / nm	τ_2 / ms	R_2 / %	d_2 / nm
1 nM	0.290	85.4	10.1	3.42	14.6	119
5 nM	0.265	67.6	9.20	2.04	32.4	70.9
10 nM	0.275	62.6	9.60	1.39	37.4	48.4
50 nM	0.290	58.5	10.1	1.98	41.5	68.8

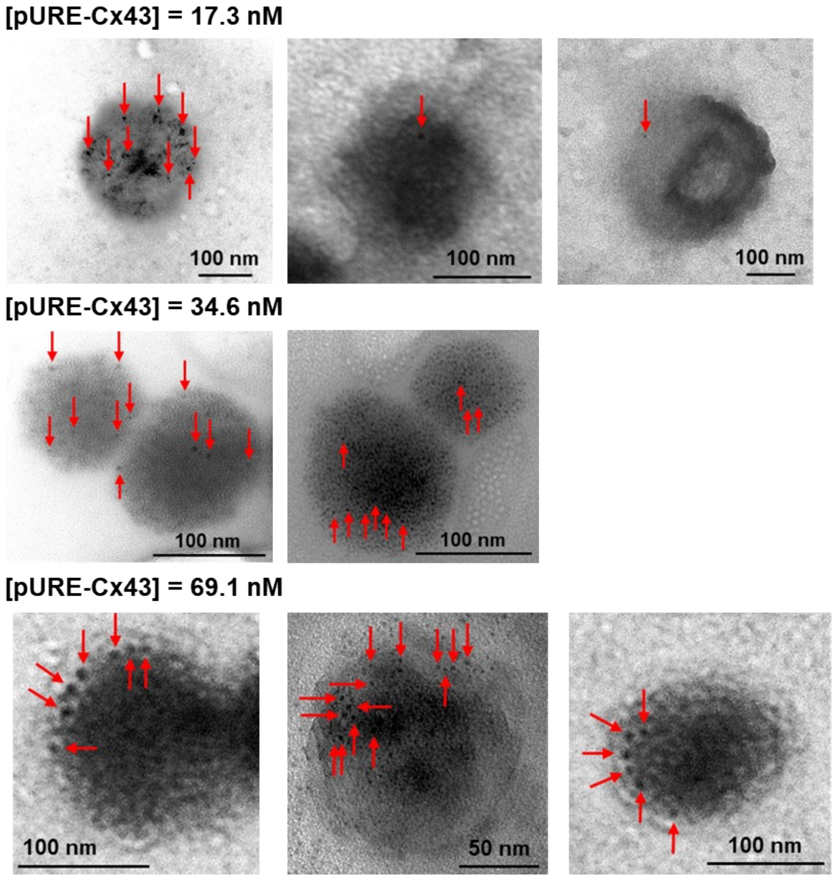

Figure S9. Density of gold nanoparticle-labelled secondary antibody (14.8 nM) bound to Cx43-embedded viral replica surface ([pURE-Cx43] = 17.3, 34.6, 69.1 nM, [anti-Cx43 antibody] = 42 nM). TEM samples were stained with EM stainer. Red arrows indicate the gold nanoparticle-labelled secondary antibody.

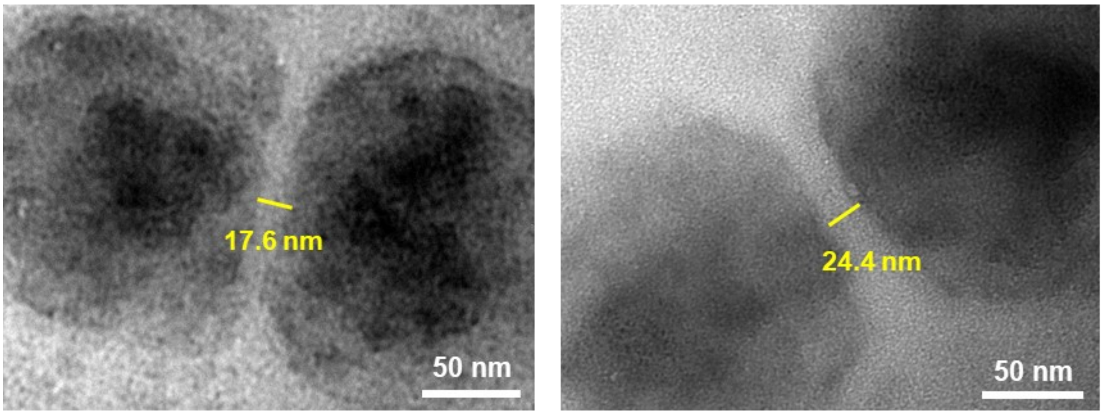

Figure S10. TEM images of gap junction between Cx43-embedded viral replica. The sample was stained with EM stainer.

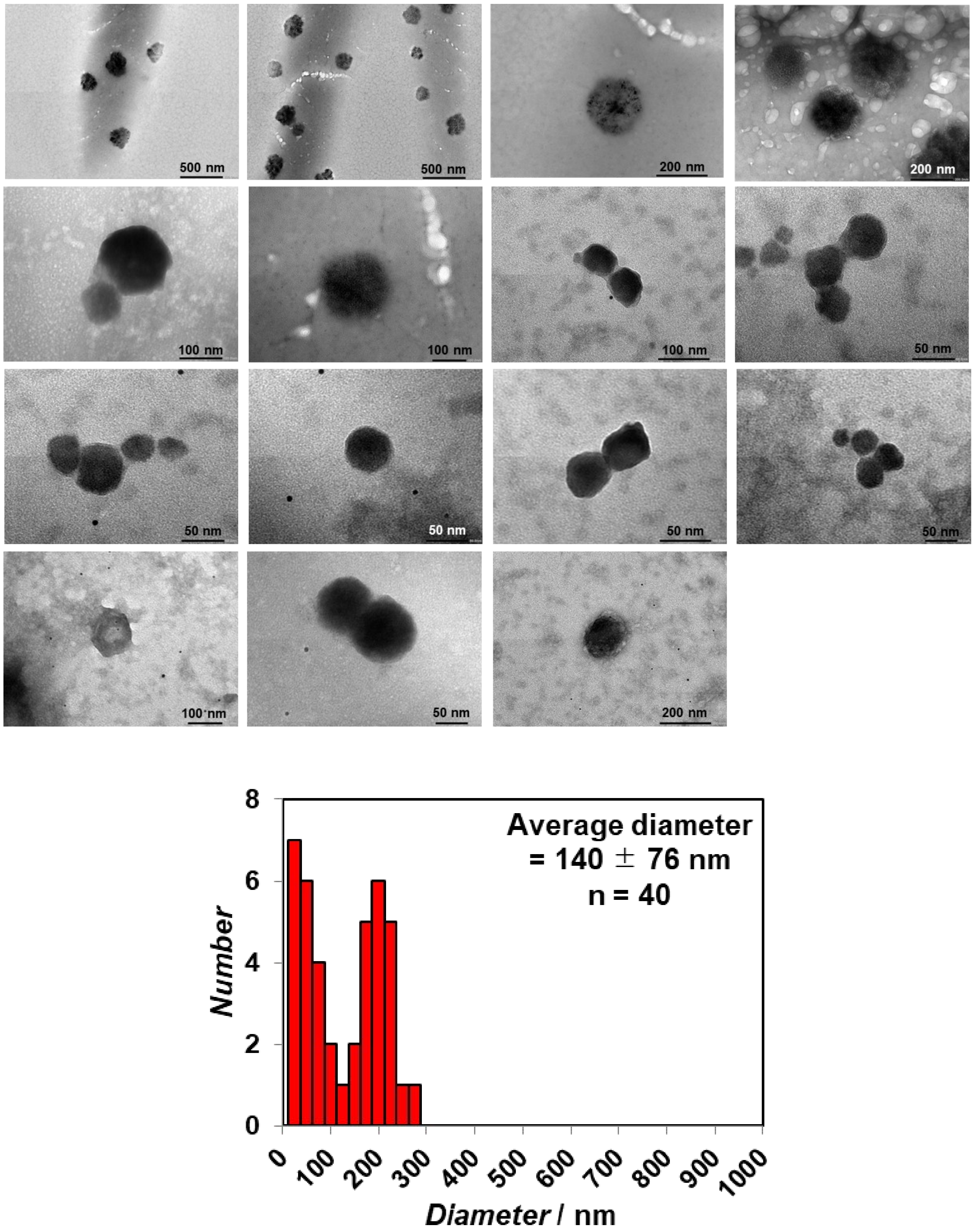

Figure S11. TEM images of anti-Cx43 antibody (42 nM) and gold nanoparticle-labelled secondary antibody (14.8 nM) in the presence of Cx43-embedded viral replica ([β-annulus-EE] = 4 μM, [DOTAP] = 12 μM, [DOPC] = 120 μM) and size distribution. Cx43 was expressed from 17.3 nM pURE-Cx43 using PURE system. The sample was stained with EM stainer.

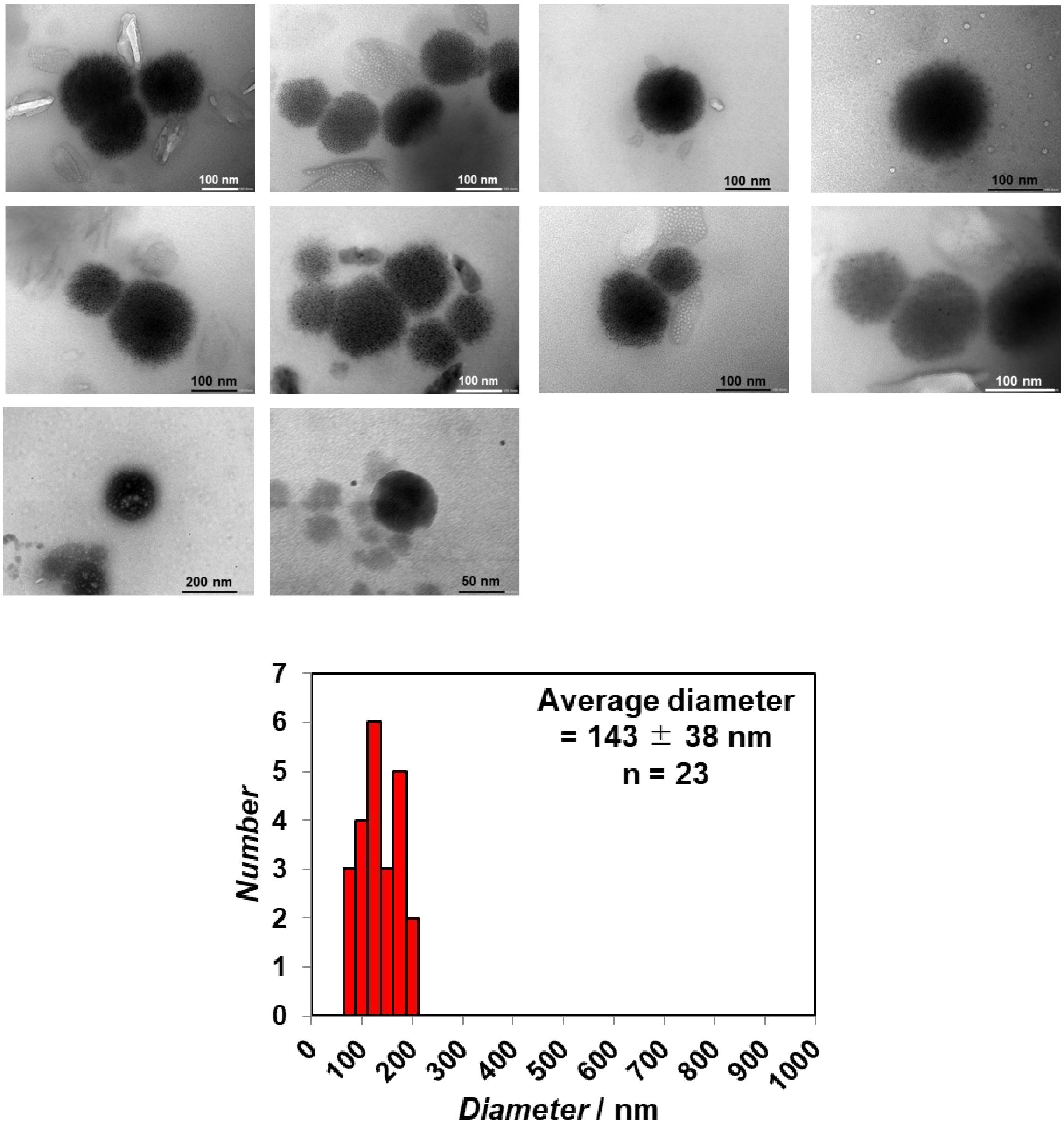

Figure S12. TEM images of anti-Cx43 antibody (42 nM) and gold nanoparticle-labelled secondary antibody (14.8 nM) in the presence of the Cx43-embedded viral replica ([β-annulus-EE] = 4 μM, [DOTAP] = 12 μM, [DOPC] = 120 μM) and size distribution. Cx43 was expressed from 34.6 nM pURE-Cx43 using PURE system. The sample was stained with EM stainer.

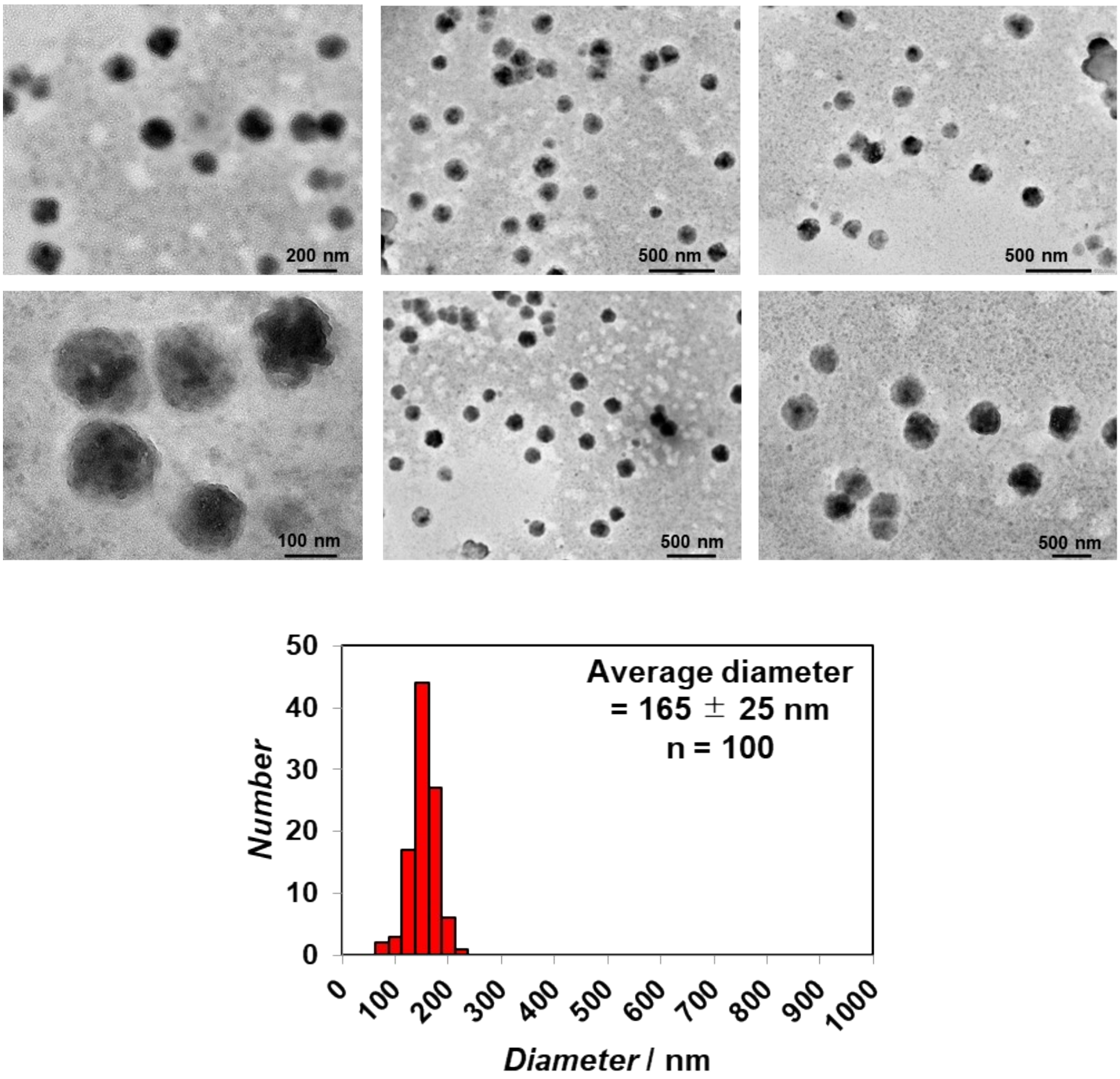

Figure S13. TEM images of anti-Cx43 antibody (42 nM) and gold nanoparticle-labelled secondary antibody (14.8 nM) in the presence of the Cx43-embedded viral replica ([β-annulus-EE] = 4 μM, [DOTAP] = 12 μM, [DOPC] = 120 μM) and size distribution. Cx43 was expressed from 69.1 nM pURE-Cx43 using PURE system. The sample was stained with EM stainer.

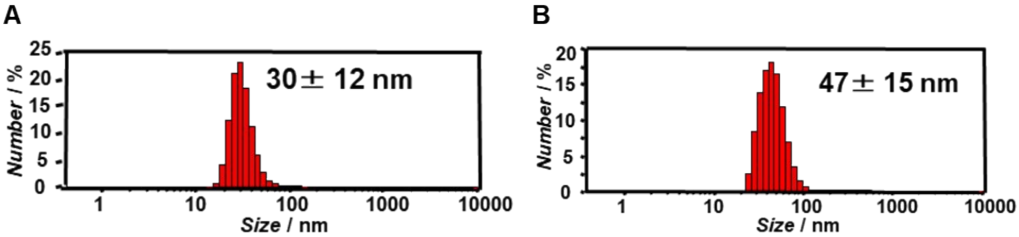

Figure S14. Size distributions obtained from DLS of (A) artificial viral capsid and (B) enveloped capsid encapsulated with 5-TMR ([β-annulus-EE] = 50 μM, [DOTAP] = 150 μM, [DOPC] = 1500 μM, [5-TMR] = 8.5 μM).

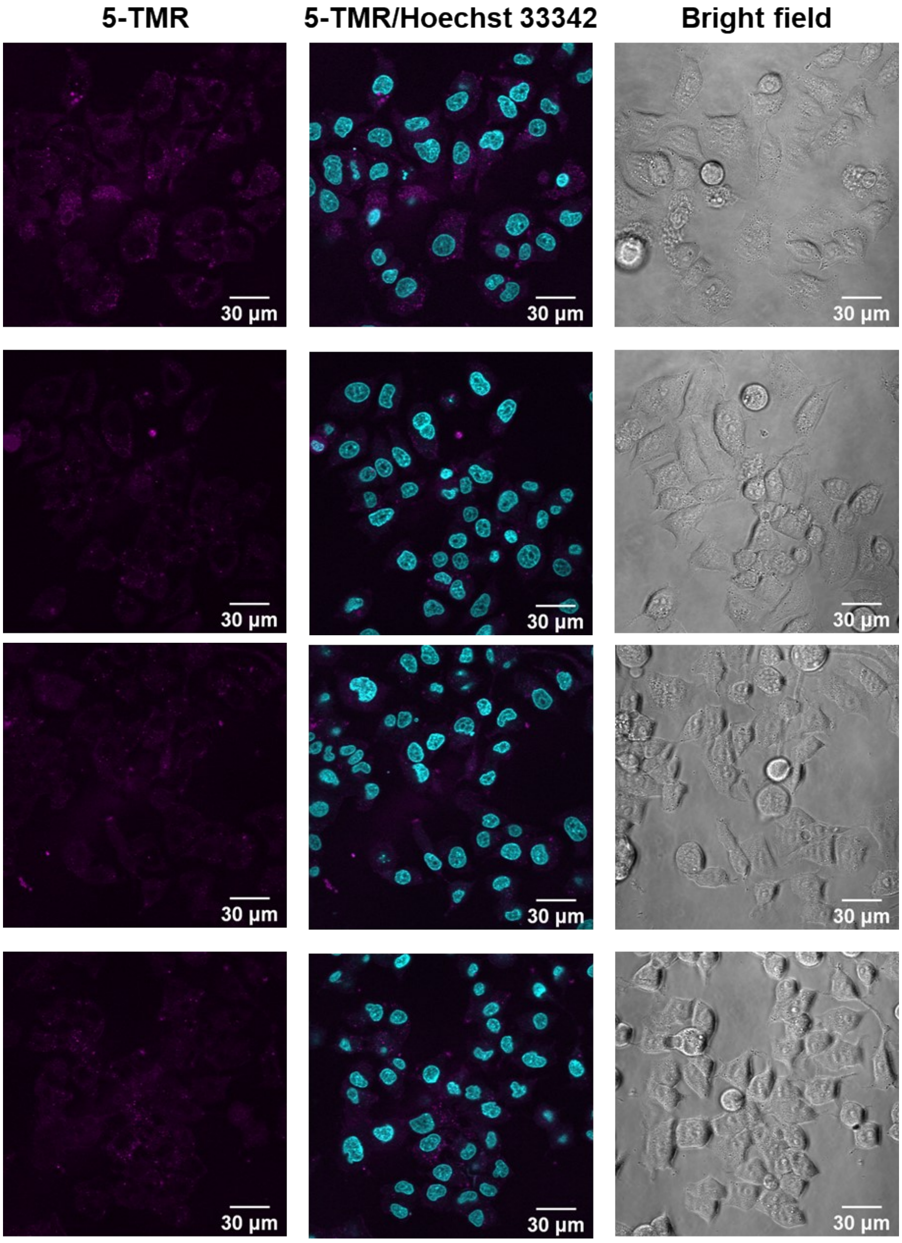

Figure S15. (A) CLSM images of HepG2 cells incubated with 5-TMR-encapsulated Cx43-embedded viral replica ([β-annulus-EE] = 10 μM, [DOTAP] = 30 μM, [DOPC] = 300 μM, [5-TMR] = 1.7 μM, [pURE-Cx43] = 17.3 nM). Channels for 5-TMR (magenta), Hoechst 33342 (cyan), bright field for CLSM images.

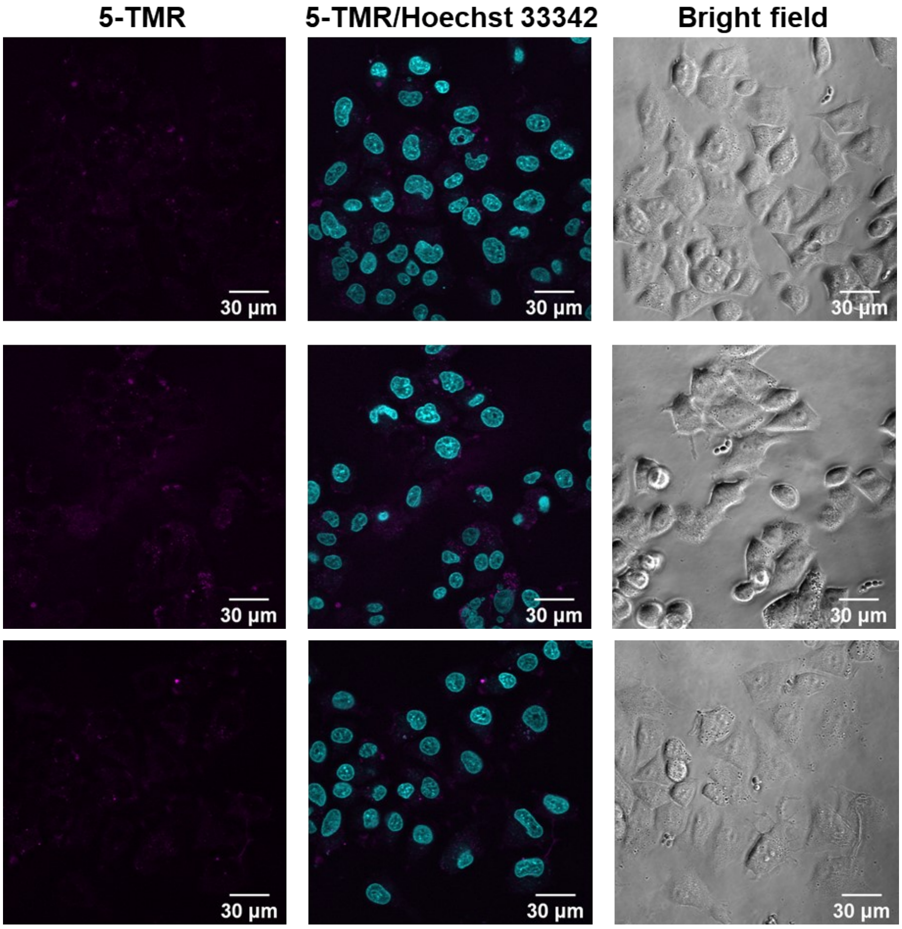

Figure S16. (A) CLSM images of HepG2 cells incubated with 5-TMR-encapsulated enveloped capsid without Cx43 ([β-annulus-EE] = 10 μM, [DOTAP] = 30 μM, [DOPC] = 300 μM, [5-TMR] = 1.7 μM). Channels for 5-TMR (magenta), Hoechst 33342 (cyan), bright field for CLSM images.
